# Comment on: “Sirolimus versus mycophenolate mofetil for the treatment of lupus nephritis: Results from a real-world CSTAR cohort study”

**DOI:** 10.1515/rir-2026-0009

**Published:** 2026-03-30

**Authors:** Umut Bakay

**Affiliations:** Department of Rheumatology, Denizli State Hospital, Denizli, Turkey

Dear Editor,

We read with great interest the article by Bai *et al*.^[1]^ comparing sirolimus and mycophenolate mofetil (MMF) for lupus nephritis (LN) using the extensive Chinese SLE Treatment and Research Group (CSTAR) real-world registry. The authors provide valuable data suggesting comparable renal and clinical outcomes between sirolimus and the current standard of care (SoC), with additional serological improvements, reinforcing sirolimus as a promising candidate. The study also acknowledges several methodological constraints, including the limited sample size and follow-up duration, which helps frame the interpretation of the results. However, several methodological aspects warrant careful consideration to contextualize these findings.

First, the small matched cohort (*n* = 53 per arm) likely results in low statistical power, potentially below 80% to detect clinically meaningful differences in remission rates (*e.g*., a 15%–20% absolute difference), which may underlie the nonsignificant *P* values reported. This range aligns with effect-size assumptions frequently used in LN phase 3 trials such as phase 3 voclosporin trial in lupus nephritis (AURORA-1), where differences of approximately 15%–20% in complete remission are required to achieve adequate power.^[2]^ Given that LN outcomes often depend on histopathological subtype, the relatively low renal biopsy rate (~25%) may obscure class-specific treatment responses. Stratified analyses by International Society of Nephrology/Renal Pathology Society classification (ISN/RPS) class or chronicity index could strengthen the conclusions regarding non-inferiority to mycophenolate mofetil (MMF).

Furthermore, the high attrition rate at the 12-month follow-up (approximately 75% of the initial cohort), while common in real-world studies, introduces potential bias and limits the reliability of the long-term conclusions. The reduced number of evaluable patients (13 in the sirolimus group and 14 in the MMF group) may particularly affect the interpretation of durability in renal remission and serologic normalization.

Second, although sirolimus demonstrated favorable complement normalization, the significant imbalance in reported adverse events (10 *vs*. 1), particularly the three cases of “mild renal insufficiency” in the sirolimus group, raises safety concerns. Clarifying whether these events were attributed to drug toxicity, background systemic lupus erythematosus (SLE) activity, or comorbidities is crucial for a comprehensive risk–benefit assessment. This clarification is particularly relevant given the known metabolic and hematologic effects associated with mTOR inhibition.^[3,4]^

Third, the treat-to-target (T2T) approach applied in this study is commendable and aligns with current European Alliance of Associations for Rheumatology (EULAR) recommendations.^[5]^ Achieving lupus low disease activity state (LLDAS)/remission in only ~60% of patients at 12 months reflects ongoing disease activity. The long-term ability of sirolimus to maintain stable renal and serological remission beyond one year, a key prognostic indicator, is still uncertain.

Overall, this study contributes valuable real-world evidence supporting sirolimus as a potential therapeutic option for LN, particularly for patients refractory or intolerant to MMF. Nevertheless, these observations highlight the need for randomized controlled trials with biopsy-proven cohorts, adequate sample size, and extended follow-up to robustly determine the long-term efficacy–safety profile of sirolimus within modern T2T frameworks.
